# Supplement to the Introduction to the Atomic Theory. Prefaced by Some Remarks on the Projected Reforms in Academical Education

**Published:** 1840-10

**Authors:** 


### Art. XVII.

*Supplement to the Introduction to the Atomic Theory; comprehending a Sketch of certain Opinions and Discoveries bearing upon the general Principles of Chemical Philosophy, which have been brought into notice since the publication of that work. Prefaced by some Remarks on the projected Reforms in Academical Education.* By Charles Daubeny, m.d. f.r.s. &c. &c. Professor of Chemistry and Botany in the University of Oxford.—*London*, 1840. 8vo, pp. 62.

Our present notice of Dr. Daubeny's most excellent and interesting pamphlet will be confined to the prefatory remarks, which so completely harmonize with the views we have recently expressed in our articles on education, that we cannot deny ourselves the pleasure of sanctioning these by the weight of Dr. Daubeny's authority. In our next Number, we shall employ his sketch of opinions and discoveries, as the basis of a general review of the present state and prospects of chemical philosophy.

"He must indeed be blind to the signs of the times," says Dr. Daubeny, "as well as to the current of academical feeling, who does not anticipate that the period is approaching, at which the system pursued at this university (Oxford), will undergo some considerable modification. There is, indeed, no predicting how long a time it may require to surmount the practical difficulties which serve to arrest the movement; but...... I cannot but feel convinced that the demand existing amongst all classes for that description of knowledge which involves some acquaintance with the truths of physical science, will, sooner or later, react upon the university, and impart a new character to the studies of those whom she sends forth to answer the urgent call for national instruction. Already this tendency of the public mind has begun to manifest itself here, by the measures for the encouragement of professional lectures which have been submitted to convocation, and which are known to have been rejected by that body, only on the alleged ground of the insufficiency of the proposed scheme to effect the desired end." (Preface, p. vii.)

We shall, indeed, rejoice when the gigantic means and influence of this noble university are employed more in accordance with the spirit of the age, and consequently with more benefit to the British public than at present. We do not go so far as to assert (with some) that the universities ought to *lead* rather than to *follow* in the path of educational improvement; for, considering the irreparable evils which would result from any false move when made on a scale of such magnitude, we think that it is the duty of those who govern those institutions, to adopt no step that is not sanctioned by experience. But it is equally their duty not to lag too far in the rear; and not to persist in compelling the student of the nineteenth century to pass through a course of education framed upon the model of the sixteenth. We are satisfied that, by admitting into their curriculum those branches of knowledge of which every man who mixes with the world now feels the want, the cause of ancient learning (whose value we by no means wish to depreciate) will not really suffer; for there will always (such is the variety of tastes in the human mind) be found enough to pursue it from choice; and the secession of those who now devote themselves to it from temporary emulation, or labour at it under compulsion, will be no real loss. In the following sound remarks on the relative value to the student of different branches of scientific knowledge, our principle of advancing from the *general* to the *special* will be at once recognized:

"It is too much the custom, even amongst the advocates of modern science, to regard its several departments as if they were placed on the same relative level, considering them all indeed as deserving the attention of men of education, —but all as equally belonging to the superstructure, of which classical learning alone is to supply the foundation. Of the utility of the latter, as constituting an essential part of primary education, I wish not to express a doubt; but I am at the same time of opinion, that the above-mentioned mode of considering the physical sciences has weakened the cause of those who advocate an extension of our system; and, instead of tending to advance in public estimation any one of its departments, has lowered them all to the same standard as that which the least important of their number is conceived to occupy. It is quite true indeed that there is no one branch of knowledge taught in this university, to which the attention of the student might not profitably be directed, or which if cultivated in a proper spirit, would not tend to enlarge the range of his ideas and to increase the sphere of his utility. But there is still, I humbly conceive, a distinction to be made in favour of those studies, which constitute the grammar to every other kind of natural knowledge, and without some acquaintance with which the student must be content to remain in the same state of ignorance, as to the causes of the commonest phenomena of the material world, which he would experience with respect to the moral if destitute of the ordinary rudiments of scholarship. In such a predicament that individual must feel himself, however high his attainments in other respects may be, who enters the world profoundly ignorant of the great physical and chemical laws of matter—that is, of the nature of those forces which operate on all bodies whatsoever, and of those distinctive properties, which characterize the substances most familiar to him, and most subservient to the common purposes of life." (Preface, p. x.)

Dr. Daubeny then goes on to argue that, though particular branches of scientific knowledge may have their special use in after-life,—as, for instance, natural history to the country clergyman, or modern history or political economy to the legislator, or the principles of jurisprudence to the country magistrate,—these studies are entered upon under a great disadvantage, without such previous attention to the *most general* branches of knowledge, as shall have afforded useful preliminary information, and shall have counteracted "that narrowness of mind which is apt to be engendered by an exclusive addiction to one particular line of studies."

"I do not then assert," continues Dr. Daubeny, "that mechanical or chemical philosophy hold in themselves a higher place than any of the above-mentioned studies, but I would merely suggest that such a knowledge of either as is requisite for duly comprehending the truths of other natural sciences—such a degree of elementary information respecting them as is assumed in the very explanations offered of the leading phenomena of the latter, ought to have a prior claim to the attention of the student, and be ranked rather as *parts of the foundation* of a liberal education, than as studies which may be advantageously ingrafted upon it." (p. xii.)

With this last opinion we most heartily coincide; and it gives us the most sincere pleasure to meet with it in the writings of an Oxford professor, and to learn from him that he is not solitary in his feeling on the subject, and that there is even a faint probability of a speedy improvement.

